# A multimodal characterization of cardiopulmonary resuscitation-associated lung edema

**DOI:** 10.1186/s40635-024-00680-1

**Published:** 2024-10-09

**Authors:** Aurora Magliocca, Davide Zani, Donatella De Zani, Valentina Castagna, Giulia Merigo, Daria De Giorgio, Francesca Fumagalli, Vanessa Zambelli, Antonio Boccardo, Davide Pravettoni, Giacomo Bellani, Jean Christophe Richard, Giacomo Grasselli, Emanuele Rezoagli, Giuseppe Ristagno

**Affiliations:** 1 Department of Pathophysiology and Transplants, University of Milan, Milan, Italy; 2 Department of Veterinary Medicine and Animal Sciences, University of Milan, Milan, Italy; 3 Department of Biomedical Sciences for Health, University of Milan, Milan, Italy; 4 Department of Acute Brain and Cardiovascular Injury, Istituto di Ricerche Farmacologiche Mario Negri IRCCS, Milan, Italy; 5 School of Medicine and Surgery, University of Milan-Bicocca, Monza, Italy; 6 CISMed - Centre for Medical Sciences, University of Trento, Trento, Italy; 7 Anesthesia and Intensive Care, Santa Chiara Hospital, Trento, Italy; 8 Ventilation Laboratory (Vent’Lab), Medical Intensive Care Unit (ICU), Angers University Hospital, Angers, France; 9 Med(2)Lab, Air Liquide Medical Systems, Antony, France; 10 Medical Intensive Care Unit (ICU), Angers University Hospital, Angers, France; 11 Department of Anesthesia, Critical Care and Emergency, Fondazione IRCCS Ca’ Granda Ospedale Maggiore Policlinico, Via Della Commenda, 16, 20122 Milan, Italy; 12 Department of Emergency and Intensive Care, Fondazione IRCCS San Gerardo dei Tintori Hospital, Monza, Italy

## Abstract

**Background:**

Cardiopulmonary resuscitation-associated lung edema (CRALE) is a phenomenon that has been recently reported in both experimental and out-of-hospital cardiac arrest patients.

We aimed to explore the respiratory and cardiovascular pathophysiology of CRALE in an experimental model of cardiac arrest undergoing prolonged manual and mechanical chest compression (CC). Oxygen delivery achieved during mechanical or manual CC were also investigated as a secondary aim, to describe CRALE evolution under different hemodynamic supports generated during CPR.

**Methods:**

Ventricular fibrillation (VF) was induced and left untreated for 5 min prior to begin cardiopulmonary resuscitation (CPR), including CC, ventilation with oxygen, epinephrine administration and defibrillation. Continuous mechanical and manual CC was performed alternating one of the two strategies every 5 min for a total of 25 min. Unsynchronized mechanical ventilation was resumed simultaneously to CC. A lung computed tomography (CT) was performed at baseline and 1 h after return of spontaneous circulation (ROSC) in surviving animals. Partitioned respiratory mechanics, gas exchange, hemodynamics, and oxygen delivery were evaluated during the experimental study at different timepoints. Lung histopathology was performed.

**Results:**

After 25 min of CPR, a marked decrease of the respiratory system compliance with reduced oxygenation and CO_2_ elimination were observed in all animals. The worsening of the respiratory system compliance was driven by a significant decrease in lung compliance. The presence of CRALE was confirmed by an increased lung weight and a reduced lung aeration at the lung CT, together with a high lung wet-to-dry ratio and reduced airspace at histology. The average change in esophageal pressure during the 25-min CPR highly correlated with the severity of CRALE, i.e., lung weight increase.

**Conclusions:**

In this porcine model of cardiac arrest followed by a 25-min interval of CPR with mechanical and manual CC, CRALE was consistently present and was characterized by lung inhomogeneity with alveolar tissue and hemorrhage replacing alveolar airspace. Despite mechanical CPR is associated with a more severe CRALE, the higher cardiac output generated by the mechanical compression ultimately accounted for a greater oxygen delivery. Whether specific ventilation strategies might prevent CRALE while preserving hemodynamics remains to be proved.

## Introduction

Cardiopulmonary resuscitation (CPR) is a life-saving intervention in cardiac arrest patients [1]. While high-quality performed chest compression (CC) showed to positively affect clinical outcomes [2, 3], the understanding of its impact on the respiratory system function during CPR and after resuscitation is still limited.

Recently, a growing attention has been drawn to pulmonary complications following CPR, highlighting a potential contribution to unfavorable outcomes. Notably, up to 50% of cardiac arrest survivors meet the criteria for acute respiratory distress syndrome (ARDS) within 48 h from hospital admission, a condition associated with high hospital mortality and poor neurological prognosis [4].

Cardiopulmonary resuscitation-associated lung edema (CRALE) is a specific form of lung damage that has been recently described both experimentally and clinically after cardiac arrest and CPR [5, 6]. CRALE is characterized by increased lung weight and density with reduced aeration, as measured by computed tomographic (CT) scan analysis. It is associated with decreased respiratory system compliance and gas exchange and appears to be more prominent after prolonged mechanical CC as compared with manual CC [5]. Thus, the imputed primary cause of CRALE might be a dynamic reduction of lung volumes together with wide swings of intrathoracic pressure occurring during vigorous CC.

However, the precise pathophysiology underlying the development of CRALE and the cardio-respiratory coupling during CPR, including whether CRALE impacts oxygen transport to the tissues, remains unclear. Furthermore, a multimodal assessment of CRALE is needed to allow identification and recognition of a clinical condition potentially underdiagnosed that deserves attention in the post-resuscitation phase.

The primary aim of this study was to provide a comprehensive CRALE characterization in a porcine model of cardiac arrest with prolonged CPR, encompassing partitioned respiratory mechanics, gas exchange, hemodynamics, chest CT scan, and histological analyses.

As secondary aim, the differences in gas exchanges and in amount of oxygen delivery achieved during mechanical or manual CC were also investigated to better describe the evolution of CRALE under different hemodynamic supports produced during CPR. We hypothesized that CRALE would develop consistently after prolonged CPR. We further hypothesized that oxygen delivery during mechanical CC would be lower compared to manual CC due to a worse oxygenation, i.e., a more severe CRALE, despite an expected higher cardiac output.

## Methods

The experiments were performed in an established model of cardiac arrest and CPR in the pig [5, 7]. We performed CPR alternating mechanical and manual CC to resemble a real-life clinical scenario including basic and advanced life support and hospital transportation, in which may be required to pause mechanical CC.

### Ethics statement

All procedures involving animals and their care conformed to national and international laws and policies. The approval of the study was obtained from the institutional review board and governmental Institution (Ministry of Health approval no. 461/2021-PR). Animal facilities meet international standards and are regularly checked by a certified veterinarian responsible for health monitoring, animal welfare supervision, experimental protocols and review of procedures.

### Animal preparation

Ten male domestic swine (33 ± 9 kg) were fasted the night before the experiments, with free access to water. Anesthesia was induced by intramuscular injection of ketamine (20 mg/kg) followed by intravenous administration of propofol (2 mg/kg) and fentanyl (3 μg/kg) through an ear vein access. Anesthesia was then maintained with a continuous intravenous infusion of propofol (4–8 mg/kg/h). A cuffed endotracheal tube was placed and animals were mechanically ventilated in volume-controlled mode with a tidal volume of 10 mL/kg, a fraction of inspired oxygen (FiO_2_) of 0.21, a positive-end expiratory pressure of 5 cmH_2_O, and a I:E 1:2 (Bellavista 1000, IMT Medical, Switzerland). The respiratory rate was adjusted to maintain the end-tidal partial pressure of carbon dioxide (EtCO_2_) between 35 and 40 mmHg, using an infrared capnometer (X-Series defibrillator ZOLL Med. Corp. Chelmsford, MA, USA). To measure aortic pressure, a fluid-filled 7F catheter was advanced from the right femoral artery into the thoracic aorta. To measure right atrial and pulmonary artery pressure, core temperature, and cardiac output, a 7F pentalumen thermodilution catheter was advanced from the right femoral vein into the pulmonary artery. For inducing ventricular fibrillation (VF), a 5F pacing catheter was advanced from the right external jugular vein into the right ventricle. An esophageal balloon (NutriVent^™^, Sidam s.r.l., Mirandola, Italy) was inserted to measure esophageal pressure (Pes).The position of all catheters was confirmed by characteristic pressure morphology and/or fluoroscopy. Frontal plane ECG was recorded.

### Experimental procedure

**Fig. 1:**
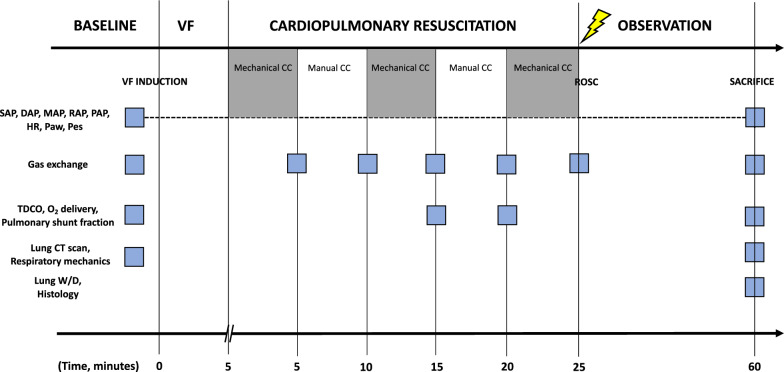
Timeline of the experimental protocol

The timeline of the experiments is detailed in Fig. 1. Animals underwent a chest CT scan before the induction of cardiac arrest. VF was then induced delivering 1–2 mA alternating current to the endocardium of the right ventricle [7]. Mechanical ventilation was discontinued after onset of VF and the endotracheal tube was left open to room air. After 5 min of untreated VF, continuous mechanical and manual CC was started and performed alternating one of the two strategies every 5 min for a total of 25 min. Unsynchronized mechanical ventilation was resumed simultaneously to CC, with the following parameters: volume-controlled mode with tidal volume of 500 ml, respiratory rate of 10 breaths/min, inspiratory to expiratory ratio (I:E) 1 to 1, FiO_2_ of 1.0 and zero positive end-expiratory pressure (ZEEP) (Bellavista 1000, IMT Medical, Switzerland). Every 5 min during CPR, epinephrine (1 mg) was administered via the right atrium, while arterial blood gasses were obtained. After 15 and 20 min of CPR, venous mixed samples were also obtained and thermodilution cardiac output was assessed. Manual CC was provided in accordance to international CPR guidelines, as previously reported [1, 7]. Mechanical CC was delivered by the LUCAS® 3.0 chest compression system (Stryker/Jolife AB, Lund, Physio-Control, Sweden), which delivers continuous CC (rate: 102 ± 2 per min; depth: 53 ± 2 mm; duty cycle: 50 ± 5%). After the 25-min interval of CPR, defibrillation was attempted with a single biphasic 150-Joule shock, using an X-Series defibrillator (ZOLL Med. Corp. Chelmsford, MA, USA). Return of spontaneous circulation (ROSC) was defined as the presence of sinus rhythm with a mean arterial pressure (MAP) of more than 60 mmHg. If ROSC was not achieved, CPR was resumed and continued for 1 min prior to a subsequent defibrillation with an escalating energy strategy (150-200-J). If VF reoccurred after ROSC, an immediate defibrillation was delivered. The same resuscitation protocol was continued until successful resuscitation or for a maximum of 5 additional min. Post ROSC mechanical ventilation was provided using volume-controlled mode with the following parameters: tidal volume 10 mL/kg, positive-end expiratory pressure of 5 cmH_2_O, I:E 1:2, a fraction of inspired oxygen (FiO_2_) of 0.3. One hour after ROSC, another lung CT scan was performed in surviving animals. Animals were then euthanized painlessly with an intravenous injection of 1 mL/10 kg Tanax, a solution of three drugs: embutramide, mebezonium iodide, tetracaine and exsanguinated through an incision in the inferior vena cava. Lungs were then harvested.

### Measurements

Hemodynamics, ECG, airway pressure (Paw) and esophageal pressure (Pes) were recorded continuously on a personal computer-based acquisition system (Labchart 8.0, Powerlab ADInstruments). The coronary perfusion pressure was computed from the differences in time-coincident diastolic aortic pressure and right atrial pressure. Cardiac output was measured by thermodilution (COM-2, Baxter International Inc).

Paw was measured at the airway opening. Pes measure served to separate the chest wall from the lung contribution to respiratory system mechanics and to describe the variation of esophageal pressure during CC (ΔPes). An occlusion test was performed by applying manual compression on the chest during airway occlusion to ensure proper placement of the esophageal catheter. The acceptable range of ΔPes/ΔPaw ratio during the occlusion test was 10–20% (i.e., from 0.8 to 1.2) [8, 9]. Every 5 min of CPR, the minimum, the maximum, and the swing in Pes were computed and ΔPes was reported. Compliance of the respiratory system, lung and chest wall was assessed at baseline and after CPR using the esophageal catheter [10]. Driving pressure (Plateau pressure-PEEP) of respiratory system, lung, and chest wall was assessed at baseline and after CPR by performing an inspiratory and expiratory occlusion maneuver of at least 5 s.

Thermodilution cardiac output, arterial, and mixed venous blood gas analyses were assessed at baseline, during mechanical and manual CC cycles of CPR, respectively at minute 15 and 20 of CC, and 1-h post-ROSC. Oxygen delivery (DO_2_) was calculated as cardiac output x arterial oxygen content (CaO_2_). Oxygen uptake (VO_2_) was calculated as cardiac output × arterio-venous oxygen content difference (CaO_2_-CvO_2_). Pulmonary shunt fraction was calculated using Berggren equation: Qs/Qt = (CcO_2_ − CaO_2_)/(CcO_2_ − CvO_2_) [11, 12]. Blood gas analyses were assessed with i-STAT System (Abbott Laboratories, Princeton, NJ).

### Necropsy

Lungs were excised en bloc, dissected from the trachea, main bronchi and hilar lymph nodes, and weighted ex-vivo within 30 min from chest CT scan.

Subsequently, bronchoalveolar lavage (BAL) was performed in the right lung, by clamping the left bronchus. Lavage was performed three times, with 10 mL of lavage solution (PBS). The BAL samples obtained were then centrifuged for 10 min, 1500 rpm, 4 °C. BAL supernatant was then stored at –80 °C for subsequent analyses.

Samples for the wet-to-dry calculation were collected and processed from the left lung. Lung tissue samples were excised from the ventral and dorsal portion of the caudal left lobe, with careful dissection from surrounding tissues. Lung tissues were fixed by immersion in 10% formalin for at least 24 h and then embedded in paraffin. Five-μm thick sections were obtained and stained with hematoxylin–eosin. The extent of histologic lung damage (i.e., lung tissue versus alveolar airspace) was determined using quantitative stereological techniques as previously reported [13]. Briefly, slides prepared as described above were viewed at a 20X magnification under a microscope and using a bright field view (Leica BioSystem, Aperio digital pathologyslide scanner, ScanScope, Leica Microsystems Srl, Milano, Italy). Two fields of view from each slide were chosen at random and digitized using a digital camera (Olympus 159 BX61, Mason technologies, Milan, Italy). Images were stored in eight-bit (256 level) format. The grid reference, i.e., X and Y grid coordinates for each image was recorded by referencing the scale attached to the microscope (200 µm). A 100-point counting grid was overlaid on each image in ImageJ imaging software package (Version 1.52 k, Wayne Rashband, national Institute of Health, USA). Care was taken to ensure that the software was set to 10 × magnification. Once this grid was superimposed over the image, a touch count was performed. At each of 100 intersection points on the grid, a record was taken for each of the following: acinar tissue, nonacinar tissue, and airspace. The intra-acinar tissue was defined as all tissues within the gas exchange portion of the lung, i.e., respiratory bronchioles, alveolar ducts, alveolar sacs, and alveoli, including blood vessels contained within their walls. The intra-acinar airspace was defined as all airspaces within the lumen of respiratory bronchioles, alveolar ducts, alveolar sacs, and alveoli. Intersections of the grid were manually counted for airspace, acinar and non-acinar tissue. Non-acinar tissue was subtracted from the overall tissue to calculate the percentage of alveolar tissue.

### Chest CT scan and quantitative analysis

Chest CT was performed with a 16-slices helical CT scanner (GE Brightspeed Elite^®^, GE Healthcare, Italy) using the following parameters: 1.25 slice thickness, tube current 180 mA, tube voltage 120 kV, scan speed 1 s/rotation, 1375 pitch, and 13.75 mm/rot. The images were reconstructed with a window setting for the evaluation of the lung parenchyma (level -500 HU; width: 1700 HU) and bone tissue (level: 600 HU; width: 3000 HU). Scans were performed during breath holding at end-expiration at 5 cmH2O, with subjects in dorsal recumbency and no contrast media was used.

The OsiriX 10.0 software (Pixmeo, Switzerland) was used to perform the morphological analysis of the lung parenchyma. Quantitative analysis of lung CT scans were performed using manual segmentation in blind and published methods for volume and weight measurements [14–17].

The manual segmentation of lung parenchyma was performed with manual delineation of each lung (from the internal rib border and the external border of the mediastinum) using the mediastinal window (CT min = −250 HU, CT max =  + 150 HU). The full CT–scale window (CT min = −1000 HU, CT max =  + 1000 HU) was used instead to view bronchi, bronchioles, blood vessels, and pleural effusion allowing a more accurate identification of these structures, in order to exclude main vessels/bronchi from the segmentation [14]. Pleural effusion was also excluded from the segmentation.

Ten slices were selected: the most cranial and caudal CT-sections and eight evenly spaced CT-sections between them were analyzed. Each of these ten CT-sections was analyzed using the standard segmentation described above. Previously described densitometry method was used to assess four differently aerated lung compartments in the 10 Sects. [14–16]. The results were extrapolated to the entire lung, according to the method described by Reske et al. [17]. Identification of CRALE was performed by using previously described criteria, i.e., mean lung density ≥ -500 Hounsfield Units (HU) [5]. In each slice the lungs were divided into three sterno-vertebral areas of equal height (i.e., ventral, ventro-dorsal and dorsal) in which the number of voxels was computed within each attenuation range: hyper-aerated (−1000 to −901 HU), normally-aerated (−900 to −501 HU), poorly aerated (-500 to -101 HU) and non-aerated lung parenchyma (−100 to + 100 HU). Overall lung density was calculated as the average density of all lung slices normalized by the areas of the lung slices. The gradient of lung density was calculated as (Ventral HU-Dorsal HU)/(ventral HU)*100 [5].

### Statistical analyses

Continuous and categorical data were expressed as mean ± standard deviation and frequency (percentage), respectively. Normality of distribution of continuous variables was assessed using D’Agostino-Pearson omnibus normality test. Difference among baseline and post resuscitation was assessed using unpaired Student’s t-test or Mann–Whitney U test, according to data distribution. Differences over time were evaluated using mixed effects model with Geisser–Greenhouse correction. Mixed effect models were performed by considering time as fixed effect, subjects (i.e., pigs) were considered as a random effect. Differences between mechanics of respiratory system, chest wall and lung over time were evaluated using a repeated measurements (RM) two-way ANOVA. In the presence of RM two-way ANOVA statistical significance, a post-hoc analysis was performed by controlling the false discovery rate using a two-stage step-up method of Benjamini, Krieger and Yekutieli for multiple comparisons. Graphs were represented using mean ± standard deviation or whisker plots. Correlations between continuous data were explored using linear regression analyses. The degree of association was reported using the Pearson’s correlation coefficient (r) ranging between −1; + 1. Statistical significance was reached when the p-value < 0.05 (two-tailed). Statistical analyses were performed using STATA-14/MP (StataCorp LP, College Station, TX, USA) and GraphPad Prism 8.3.0 (GraphPad Software, San Diego, CA, USA).

## Results

**Fig. 2:**
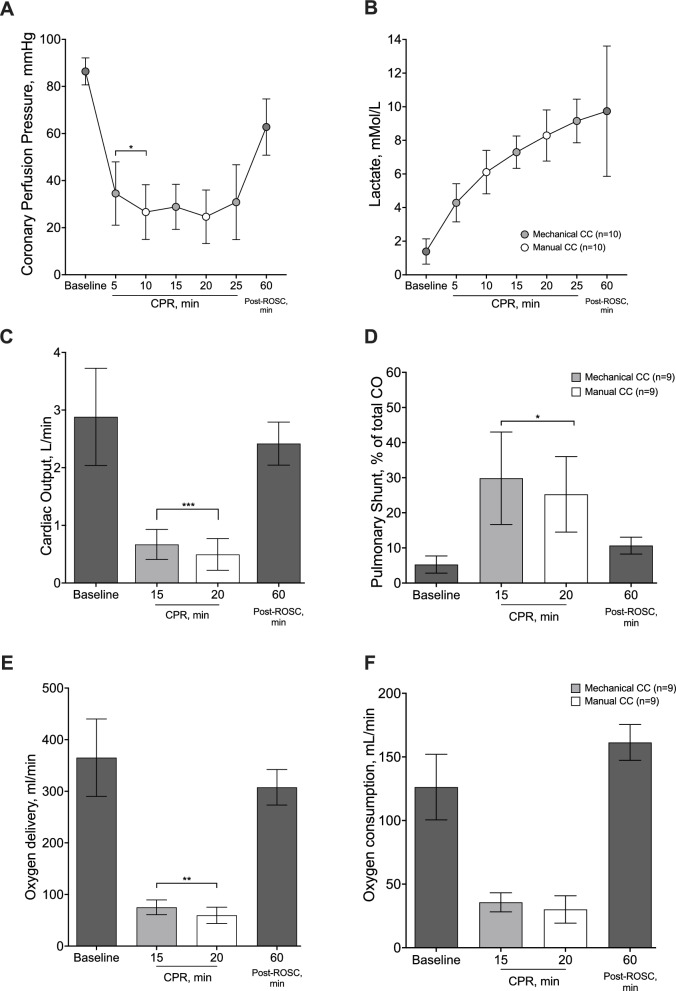
Hemodynamics and perfusion. Coronary perfusion pressure (panel **A**), lactate levels (panel **B**), cardiac output (panel **C**), pulmonary fraction of shunt (panel **D**), oxygen delivery (panel **E**) and oxygen consumption (panel **F**) over the entire experiment

**Fig. 3:**
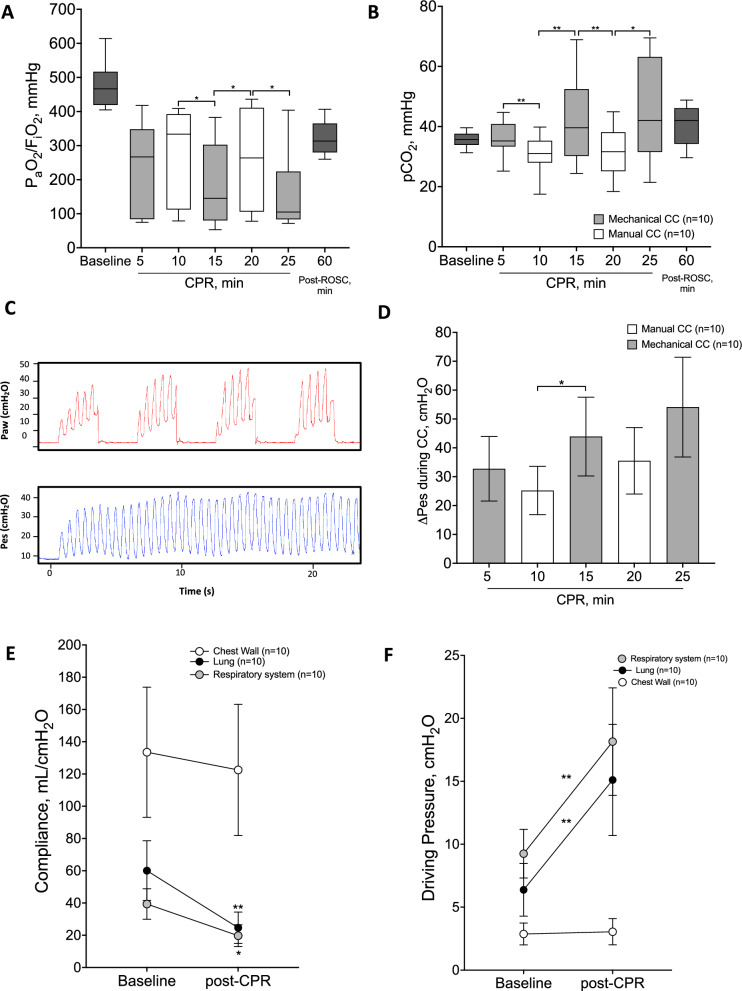
Gas exchange including oxygenation (panel **A**) and CO_2_ clearence (panel **B**) during the experiment, airway pressure and esophagel pressure waveforms during 4 consecutive breath cycles (panel **C**), change in esophageal pressure during chest compression over 24 min CPR (panel **D**) and respiratory mechanics including partitioned respiratory system compliance (panel **E**) and driving pressure (panel **F**)

**Fig. 4:**
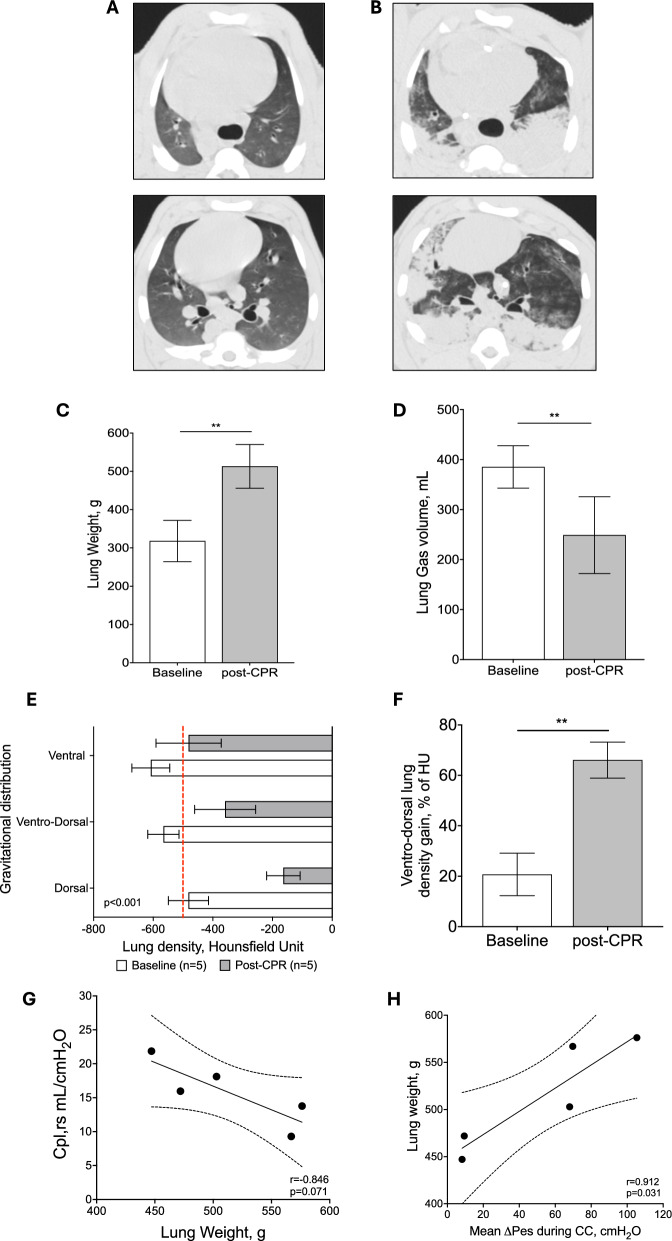
Quantitative computed tomography analyses at baseline and post-CPR. Representative axial scan images at two different levels (upper and lower) of the lungs at baseline (panel **A**) and post-CPR (panel **B**). Lung weight and lung gas volume at baseline and post-CPR (panel **C** and **D**). Lung gravitational distribution stratified in three different areas (i.e., ventral, ventro-dorsa, and dorsal) at baseline and post-CPR condition (panel **E**). The red dashed-line refers to the density threshold commonly used to separate the normally (density < −500 HU) versus poorly (density > −500 HU) aerated lung areas. In panel **F**, the gravitational change in ventro-dorsal lung density expressed as HU%. Association of lung weight with Cpl,rs (**G**) and mean Pes during CC and lung weight (**H**). In **G** and **H**, the continuous line represents the best fit line of the linear regression with the 95% confidence interval represented with dashed lines

Hemodynamic variables, arterial and mixed venous blood gas analyses and respiratory mechanics at baseline are reported in Tables 1–2 and in Figs. 2, 3, 4. Five animals (50%) achieved ROSC and survived for 1 h before euthanasia.

**Table 1:** Hemodynamic variables

	Baseline (n = 10)	Post-resuscitation (n = 5)	p
Heart rate, beats per minute	89 ± 27	139 ± 41	0.014
Systolic arterial pressure, mmHg	108 ± 23	91 ± 24	0.218
Diastolic arterial pressure, mmHg	80 ± 21	67 ± 31	0.323
Mean arterial pressure, mmHg	90 ± 25	73 ± 26	0.229
Right atrial pressure, mmHg	5 ± 2	5 ± 2	0.748
Pulmonary arterial pressure, mmHg	17 ± 7	23 ± 9	0.170
Pulmonary capillary wedge pressure, mmHg	6 ± 2	6 ± 2	0.799
Thermodilution cardiac output, L/min	3 ± 1	2.4 ± 0.4	0.173
Stroke volume, mL	35 ± 14	18 ± 4	0.019
End-tidal CO_2_, mmHg	35 ± 2	34 ± 5	0.452
Temperature, °C	37 ± 1	37 ± 1	0.989
Hb, g/L	7 ± 1	9 ± 1	0.013
Ht, %	20 ± 1	25 ± 4	0.013
Na^+^, mmol/L	141 ± 3	139 ± 2	0.428
K^+^, mmol/L	3.5 ± 0.1	4.4 ± 0.9	0.036

*End-tidal CO*_*2*_ indicates carbon dioxide at the end of expiration, *Hb* indicates hemoglobin, *Ht* indicates hematocrit

Data are expressed as mean ± SD

**Table 2:** Arterial and mixed venous blood gas analyses

	Baseline (n = 10)	Cardio-pulmonary resuscitation (n = 10)					Post-resuscitation (n = 5)	p
		5 min	10 min	15 min	20 min	25 min		
		Mechanical CC	Manual CC	Mechanical CC	Manual CC	Mechanical CC		
pH arterial	7.489 ± 0.033	7.403 ± 0.032	7.379 ± 0.037	7.244 ± 0.111	7.264 ± 0.085	7.156 ± 0.124	7.229 ± 0.149	< 0.001
PaCO_2_, mmHg	36 ± 3	36 ± 6	31 ± 7	43 ± 14	32 ± 8	46 ± 17	41 ± 7	0.037
PaO_2_, mmHg	100 ± 14	238 ± 136	269 ± 138	185 ± 122	244 ± 142	156 ± 124	96 ± 16	0.003
SaO_2_, %	98 ± 1	99 ± 2	99 ± 2	95 ± 7	98 ± 3	94 ± 5	95 ± 3	0.079
HCO_3_ arterial, mmol/L	27 ± 2	22 ± 2	18 ± 3	17 ± 4	13 ± 3	14 ± 4	18 ± 6	< 0.001
BE arterial, mmol/L	3.8 ± 3	–2.4 ± 2	–7 ± 2	–11 ± 3	–14 ± 3	–15 ± 3	–9 ± 9	< 0.001
Lactate arterial, mmol/L	1 ± 1	4 ± 1	6 ± 1	7 ± 1	8 ± 2	9 ± 1	10 ± 4	< 0.001
pH mixed venous	7.431 ± 0.02			7.090 ± 0.09	7.128 ± 0.14		7.147 ± 0.21	< 0.001
PvCO_2_, mmHg	40 ± 6			61 ± 20	57 ± 25		61 ± 15	0.040
PvO_2_, mmHg	33 ± 4			38 ± 8	39 ± 8		32 ± 5	0.139
SvO_2_, %	65 ± 5			50 ± 13	56 ± 20		46 ± 19	0.111
HCO_3_ mixed venous, mmol/L	27 ± 4			18 ± 5	17 ± 3		21 ± 5	< 0.001
BE mixed venous, mmol/L	2 ± 3			–11 ± 7	–12 ± 2		–8 ± 8	< 0.001
Lactate mixed venous, mmol/L	1 ± 1			7 ± 2	8 ± 2		9 ± 3	< 0.001
DO_2_, ml/min	344 ± 193			75 ± 40	60 ± 45		308 ± 77	< 0.001
VO_2_, ml/min	119 ± 66			36 ± 21	30 ± 30		161 ± 32	< 0.001

*BE* indicates base excess, *HCO*_*3*_ indicates bicarbonate, *PaCO*_*2*_ indicates arterial carbon dioxide partial pressure, *PaO*_*2*_ indicates arterial oxygen partial pressure, *SaO*_*2*_ indicates arterial oxygen saturation, *PvCO*_*2*_ indicates venous mixed carbon dioxide partial pressure, *PvO*_*2*_ indicates venous mixed oxygen partial pressure, *SaO*_*2*_ indicates venous mixed oxygen saturation, *DO*_*2*_ indicates oxygen delivery, *VO*_*2*_ indicates oxygen consumption

Data are expressed as mean ± SD. Differences over time were evaluated using mixed effects model with Geisser-greenhouse correction

### Hemodynamics and systemic perfusion

Coronary perfusion pressure during (p < 0.001) and after CPR (p = 0.020) was significantly lower compared to baseline (Fig. 2A). During the initial 5 min of CC, performed with a mechanical CC device, CPP was significantly higher compared to the following 5 min, with manual CC (Fig. 2A).

Arterial lactate increased steadily during CPR and up to 60 min post resuscitation, with no difference between mechanical and manual CC (p < 0.001, Table 2, Fig. 2B). Heart rate significantly increased post-ROSC as compared to baseline (p = 0.014, Table 1), together with a significant reduction in stroke volume (p = 0.008, Table 1). No significant differences in other hemodynamic variables were observed between post-ROSC and baseline (Table 1).

### Gas exchange and respiratory mechanics

The PaO_2_/FiO_2_ decreased during CPR as compared to baseline (p < 0.001). A significantly lower PaO_2_/FiO_2_ was observed at minute 15 and 25 of CC, performed with a mechanical CC device, compared to that measured at minute 10 and 20, when manual CC was delivered (Fig. 3A). The PaCO_2_ changed significantly overtime compared to baseline values (p = 0.037) showing higher values during the mechanical CC cycles as compared with the manual ones (Fig. 3B and Table 3).

**Table 3:** Lung-computed tomographic scan quantitative analysis

	Baseline (n = 5)	Post-resuscitation (n = 5)	p
Lung density, HU	–549 ± 55	–324 ± 85	0.001
Lung volume, ml	704 ± 68	762 ± 71	0.222
Lung gas volume, ml	385 ± 43	249 ± 77	0.008
Lung weight, g	318 ± 54	513 ± 57	< 0.001
Not-inflated tissue, %	0	23 ± 11	0.002
Poorly inflated lung tissue, %	26 ± 15	44 ± 11	0.021
Well-inflated lung tissue, %	74 ± 15	33 ± 17	0.002
Rib Fractures, *n* (%)			
Incomplete	0/5 (0)	5/5 (100)	
Complete compound			0.001
Dos dislodged	0/5 (0)	5/5 (100)	L 0.291
Compound dbxsuc			0.291 0.2919
Dislodged	0/5 (0)	1/5 (20)	0.292
Sternocostal dislocation	0/5 (0)	2/5 (40)	0.114

*HU* indoicates Hounsfield units

Data are expressed as mean ± SD

A representative image of the Pes and Paw waveforms during CPR is reported in Fig. 3C. The ΔPes generated by CC is reported in Fig. 3D. A significant increase of ΔPes over 25 min of CPR was observed (Fig. 3D, p = 0.034) with significantly higher values during mechanical CC cycles compared to the manual ones (Fig. 3D).

Compliance of the respiratory system decreased significantly after CPR compared to baseline values (*p* = 0.034, Fig. 3E). The change in respiratory system compliance was driven by a significant decrease in lung compliance (*p* < 0.001, Fig. 3E). Compliance of the chest wall, instead, did not change following CPR (*p* = 0.221, Fig. 3E). Lung driving pressure significantly increased after 25 min of CPR compared to baseline values (p < 0.001, Fig. 3F) while the driving pressure of the chest wall did not change (*p* = 0.314, Fig. 3F).

### Cardiac output, oxygen delivery and pulmonary shunt fraction

Cardiac output, DO_2_, VO_2_, and pulmonary shunt fraction at baseline are reported in Fig. 2 and Tables 1 and 2. Thermodilution cardiac output was markedly reduced during CPR, with values of approximately 20% of the baseline ones (p < 0.001, Fig. 2C). However, during mechanical CC, a significantly higher cardiac output was observed compared to manual CC (p < 0.001, Fig. 2C). Accordingly, DO_2_ decreased significantly during CPR (p < 0.001, Table 2) with a higher value detected during mechanical CC compared to manual CC (p < 0.001, Table 2). The pulmonary shunt fraction increased during CPR, showing higher values during mechanical CC compared to manual CC (p = 0.046, Fig. 4B).

### Quantitative analysis of lung CT scans and CRALE

Figure 4 shows representative CT images of lungs at baseline (Fig. 4A) and following 25 min of CPR (Fig. 4B). Data regarding the main quantitative CT variables are summarized in Table 3. The mean lung weight increased significantly after CPR compared to baseline (post-CPR *vs* baseline: 513 ± 57 *vs* 318 ± 54 g, p < 0.001, Table 3 and Fig. 4C). Accordingly, lung gas volume showed a significant reduction, i.e., approximately 40%, (249 *vs* 385 mL, p = 0.008, Table 3 and Fig. 4D), while lung volume did not change following CPR (762 *vs* 704, p = 0.222, Table 3). The non-inflated lung tissue was not represented at baseline while it increased up to 23% following CPR (p = 0.002, Table 3). The poorly inflated tissue significantly increased following CPR (44 *vs* 26%, p = 0.021, Table 3), while the well-inflated tissue decreased after resuscitation (74 *vs* 33%, p = 0.002, Table 3). The lung density increased following a gravitational distribution which was more pronounced after CPR compared to baseline, with a higher density observed at each gravitational level (p < 0.001, Fig. 4E). The ventro-dorsal gradient was also higher after CPR compared to baseline (66 *vs* 21%, p < 0.001, Fig. 4F).

Thus, all surviving animals after the experiment (5/5, 100%) fulfilled criteria of CRALE after 25 min of CPR. Lung weight was negatively correlated to the compliance of the respiratory system (r = −0.846, p = 0.071, Fig. 4G), and ΔPes was positively correlated to the lung weight (r = 0.912, p = 0.031, Fig. 4H). All resuscitated animals showed rib fractures, both incomplete and complete and compound. Dislodged complete fractures and sternocostal dislocation were present in respectively in 20% and 40% of animals (Table 3).

### Necropsy and histology

**Fig. 5:**
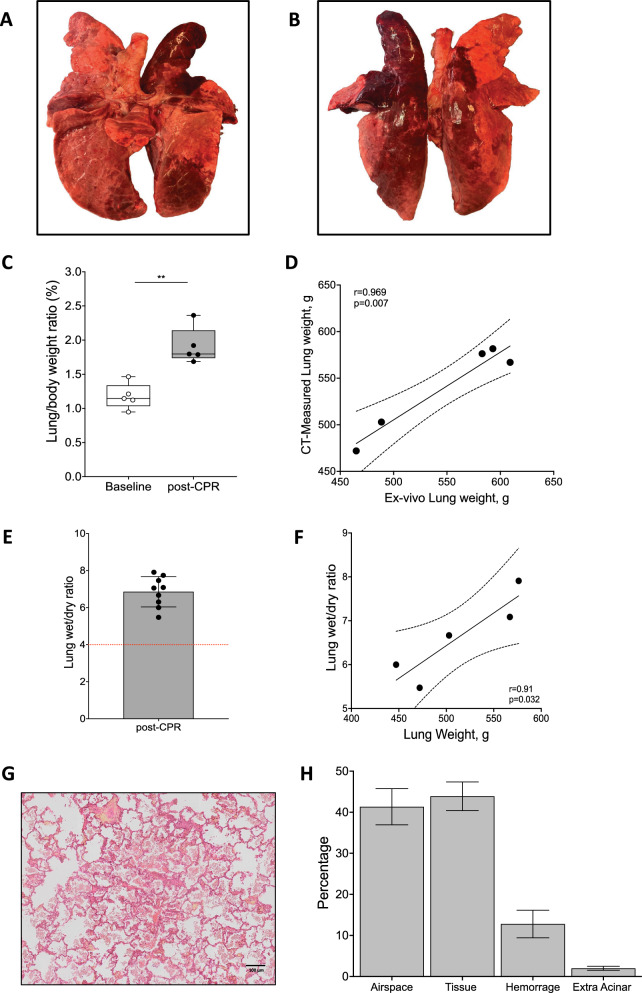
Anatomical and histological lung findings. Macroscopic lung images of an exemplary dissected lung post-CPR after the end of the experiment showing the ventral (**A**) and the dorsal (**B**) surface. In panel **C**, the comparison of the lung/body weight ratio between baseline and post-CPR. Association of lung weight measured ex-vivo and with CT scan method (**D**). In panel **E**, the lung wet-to-dry ratio post-CPR. Association of lung weight and lung wet-to-dry ratio (F). Exemplary picture of a lung histological section showing the substitution of the airspace by tissue and hemorrhagic areas (panel **G**). Histological evaluation post-CPR assessed by % of airspace, tissue, hemorrhage and extra acinar areas (panel **H**)

Figure 5 shows the main anatomical and histological findings of ventral and dorsal lungs after 25 min of CPR. Macroscopically, all pigs presented qualitative lung abnormalities including severe contusion, edema and hemorrage. Ex-vivo lung weight was 548 ± 66 g, with an excellent correlation with the CT-measured weight (r = 0.969, p = 0.007, Fig. 5D). A higher lung/body weight ratio was observed following CPR (p < 0.001, Fig. 5C) together with an abnormally high lung wet/dry ratio (Fig. 5E). Lung weight was positively correlated to lung wet/dry ratio (r = 0.910, p = 0.032, Fig. 5F). High vascular permeability was also confirmed by a significant increase in hemoglobin concentration (p = 0.013, Table 1). An exemplary image of the histological findings is reported in Fig. 5G. At histological evaluation, tissue proportion was similar to airspace proportion (44 ± 10 and 41 ± 13%, respectively), and hemorrhage was remarkably present (13 ± 10%, Fig. 5H).

## Discussion

This study provides a detailed characterization of CRALE in a porcine model of cardiac arrest and prolonged CPR performed alternating mechanical and manual CC. The effect of CPR on development of CRALE is supported by a comprehensive approach including the assessment of partitioned respiratory mechanics, gas exchange, invasive hemodynamics, and CT lung imaging data coupled with histological analyses. As anticipated, all animals developed CRALE after 25 min of CPR performed with both mechanical and manual CC. Indeed, at post-ROSC CT scan an increased lung weight and a reduced lung aeration were reported consistently. The imaging findings were supported by a marked decrease of the respiratory system compliance, a progressive reduced oxygenation and CO_2_ clearance were observed after 25 min of CPR.

### Novel findings in CRALE

This study identified key novel findings characterizing CRALE: (1) a significant decrease in lung compliance, leading to a worsening of respiratory system compliance, while chest wall compliance remained unchanged; (2) a high wet-to-dry ratio with tissue and hemorrhagic replacement of the alveolar airspace, further confirming the presence of CRALE; and (3) a higher oxygen delivery when resuscitation was performed mechanically, despite greater impairment of respiratory gas exchange during mechanical CC compared to manual.

### Advanced respiratory mechanics in CRALE

In the first translational study introducing the concept of CRALE in an experimental model of cardiac arrest, we observed that the change in intrathoracic pressure during CPR, assessed indirectly as changes in central venous pressure, was highly correlated with the density increase of the lung, as demonstrated by the lung CT [5]. Although central venous pressure changes nicely estimate the change in pleural pressure [18, 19], the gold standard technique is the esophageal pressure monitoring by an esophageal catheter [20]. In this study, we confirmed the robust association of the change in intrathoracic pressure, now explored by assessing swings in esophageal pressure, with the lung weight estimated at CT scan. This corroborated the link between vigorous CC, which usually generates a high compressing force translating into high intrathoracic pressure swings, with the development of CRALE.

In this model, the derangements of respiratory mechanics showed that CRALE was characterized by low lung compliance and high lung driving pressure while chest wall mechanics did not differ from baseline. These findings are in line with a previous observation showing that patients admitted in ICU with CRALE, clinically defined as PaO_2_/FiO_2_ ≤ 300 mm Hg at 5 cmH_2_O PEEP and bilateral infiltrates on the chest radiograph, have lower lung compliance, and end-expiratory lung volume and a higher dead space compared to patients without CRALE [6]. Indeed, lung compliance is markedly reduced in patients within a short time after cardiac arrest [21]. Dynamic reduction of lung volumes [22] together with intrathoracic airway closure [16, 23–25] occurring during CC may contribute to the unique pathophysiological condition of ventilation below the functional residual capacity observed during CPR [23]. Our model was characterized by numerous rib fractures, i.e., > 6 that however did not compromise the resuscitation success [26]. Interestingly, such a severe chest wall injury did not affect the chest wall compliance, as one could have expected following prolonged CPR [27]. Thus, CPR did not significantly impact chest wall compliance, as recently observed also in patients [6]. Differently, CPR did create an important lung injury with generation of edema and hemorrhage and consequently significant reduction in lung compliance, which ultimately represent the solely determinant for respiratory system compliance modifications after ROSC.

### Oxygen delivery and pulmonary shunt during CPR

In the current study, a considerable decline in CC-generated cardiac output was detected with values ≈20% from baseline, as previously reported [28]. However, during mechanical CC, a higher cardiac output was detected confirming the greater hemodynamic support and systemic perfusion generated by mechanical CC compared with manual CC that we previously described [9]. This higher blood flow generated by mechanical CC might have overcome the lower levels of oxygenation and the higher level of pulmonary shunt fraction observed, accounting for an overall higher oxygen delivery during mechanical CC compared to manual CC, in contrast to our initial hypothesis. These findings suggest that higher amount of oxygen is delivered to the tissues (for a given hemoglobin concentration) although in the presence of impaired ventilation/perfusion distribution during mechanical CC. Indeed, in this study, pulmonary shunt fraction increased up to 30% during mechanical CC showing that distribution of blood flow to unventilated or poorly ventilated lung units increased significantly during CPR. The higher shunt fraction detected in the mechanical CC phase of CPR could also be explained by the presence of higher cardiac output. Indeed, previous studies demonstrated a fall in pulmonary shunt fraction with reduction in cardiac output in experimental pulmonary edema [29, 30] and in ARDS patients [31]. The proposed main factors influencing this association are an increase in hypoxic pulmonary vasoconstriction and a reduction in venous oxygen content [30]. Based on these results together with our earlier studies [5], although we may argue that performing manual CC would be better in preventing and/or reducing CRALE severity compared to mechanical CC, the use of a mechanical compressor may warrant a higher cardiac output during CPR which ultimately can counterbalance the worse CRALE-dependent oxygenation. In addition, application of a small amount of PEEP may represent a valid approach, especially during mechanical compression, to prevent CC-induced airway closure, contrast lung edema development during CPR and overall enhance the amount of efficient alveolar ventilation [6, 32]. However, future studies are needed to test the above hypothesis.

Intrapulmonary shunt during CPR has been described as a consequence of inhomogeneous ventilation leading to ventral pulmonary hyperinflation and atelectasis formation in the dependent lung areas [22] aggravated by the formation of denitrogenation atelectasis [25]. Our CT findings further confirm that lung inhomogeneity is a typical feature of CRALE, as shown by the gravitational distribution with higher density in the dorsal lung regions and the significant increase in the ventro-dorsal density gain following CPR. Furthermore, at the histological evaluation, there was a higher proportion of edema within the alveolar tissue that replaces the alveolar airspace and also a significant component of alveolar hemorrhage. Although the presence of alveolar hemorrhage following CPR has been previously described [33], the causal mechanism and pathophysiological role in the evolution of CRALE have not been currently investigated.

### Future perspectives

A better comprehension of cardio-respiratory coupling during mechanical CC is highly significant, because ensuring proper ventilation in this context of extremely low cardiac output could be crucial for directing pulmonary blood flow towards lung units with a normal ventilation/perfusion ratio, allowing for the highest oxygen delivery possible. These findings raise also the question on whether an ideal ventilation strategy during CPR could be able to limit the occurrence of CRALE. In our experimental setting, during CPR, no PEEP was applied and unsynchronized mechanical ventilation was provided. However, a ventilation strategy based on application of a moderate PEEP level, [34, 35], during mechanical and/or prolonged manual CC may be beneficial as it showed to improve alveolar ventilation [34]. Therefore, whether this approach can improve ventilation homogeneity and gas exchanges while preventing dynamic reduction of lung volumes is yet to be investigated.

Finally, we believe that CRALE might represent an under-recognized clinical condition, potentially undertreated in terms of optimal and protective approaches to mechanical ventilation and monitoring of respiratory mechanics. Indeed, lung-protective ventilation following resuscitation suggests that employing a low tidal volume strategy (8 mL/kg) might enhance neurocognitive outcomes post-cardiac arrest [36]. Promoting identification of CRALE can improve ventilatory treatment strategies in the post-resuscitation phase.

### Strengths and limitations

Strengths of the study include the use of an established model of porcine cardiac arrest, with a prolonged duration of CPR and a multimodal monitoring of lung impairment including partitioned respiratory mechanics, gas exchange, lung CT and histology. Several limitations of the study should be also acknowledged. First, the fixed sequence of mechanical versus manual CC periods during CPR may have hindered our ability to evaluate the individual effects of each technique on the incidence and causes of CRALE. Second the study was limited to a single ventilation setting with no implementation of different ventilatory strategies. Third, we cannot also exclude that the high tidal volume and the absence of PEEP used during CPR accounted for the intratidal opening/closing of alveolar unit, ultimately exacerbating lung lesions and edema. However, our ventilation strategy during CC did not have any harmful hemodynamic effect. This is in line with previous experimental studies demonstrating the safety of tidal volumes up to 18 ml/kg during CPR [37]. Finally, we cannot identify the unique effects of asynchronous ventilation in the present study. However, previous observations showed that although it may increase peak airway pressures compared to synchronous ventilation [38], it did not result in increased lung injury.

## Conclusions

In a porcine model of cardiac arrest undergoing an alternating sequence of CPR by mechanical and manual CC over a prolonged period of 25 min we observed CRALE, characterized by a decrease in respiratory system compliance, that was later confirmed by a higher lung density and wet-to-dry ratio, with the presence of a significant proportion of alveolar tissue and hemorrhage replacing the airspace. Despite mechanical CPR is associated with a more severe CRALE, as represented by lower levels of oxygenation and higher levels of pulmonary shunt compared to manual CPR, the higher cardiac output generated by the mechanical compression ultimately accounted for a greater oxygen delivery. Whether specific ventilation strategies during and after CPR might prevent CRALE is a subject for future investigations.

## Data Availability

The data that support the findings of this study are not openly available and are available from the corresponding author upon reasonable request.
